# Identification and Validation of Basement Membrane Related LncRNA Signatures as a Novel Prognostic Model for Hepatocellular Carcinoma

**DOI:** 10.1007/s10528-024-10797-3

**Published:** 2024-04-29

**Authors:** Xuyang Liu, Chao Lv, Jian Zheng, Jingjing Xiao, Nan He, Jun Du, Xianwu Yang, Huajian Gu

**Affiliations:** 1 Department of Pediatric Surgery, Affiliated Hospital of Guizhou Medical University, Guiyang, China; 2 School of Clinical Medicine, Guizhou Medical University, Guiyang, China

**Keywords:** Basement membrane, Biomarkers, Hepatocellular carcinoma, Prognostic model, AC092614.1

## Abstract

Hepatocellular carcinoma (HCC) is a significant cancer with limited treatments and a poor prognosis, with the basement membrane (BM) playing a crucial role in its initiation and growth. This study utilized data from The Cancer Genome Atlas and the Gene Expression Omnibus (GEO) databases to identify basement membrane-related genes differentially expressed in HCC. Through gene co-expression analysis, BM-associated long non-coding RNAs (lncRNAs) were discovered. LncRNAs related to HCC survival were selected via univariate analysis, and a prognostic model was constructed using LASSO regression and multivariate analysis. This model effectively classified HCC patients into high and low-risk groups, uncovering significant differences in prognosis, immune response, mutation, and drug sensitivity. Six BM-related lncRNAs (GSEC, MIR4435-2HG, AC092614.1, AC127521.1, LINC02580, and AC008050.1) were validated in normal and HCC cell lines, and the key role of AC092614.1 in regulating proliferation, migration, and invasion of HCC cells in vitro was explored. This research emphasizes the prognostic and therapeutic relevance of BM-related lncRNAs in HCC, highlighting AC092614.1’s role in disease progression and as a potential target for targeted therapy.

**Supplementary Information:**

The online version contains supplementary material available at 10.1007/s10528-024-10797-3.

## Introduction

Hepatocellular carcinoma (HCC) accounts for approximately 75-85% of primary liver cancers, posing a significant health risk (Sung et al. 2021). Despite advancements in medicine and combined surgical-chemotherapy treatments, HCC’s heterogeneity and recurrence rates continue to confer a poor prognosis. The basement membrane (BM), an extracellular matrix beneath epithelial tissues, consists of collagen IV, laminins, fibronectins, and heparan sulfate proteoglycans. It plays a vital role in regulating immune cell adhesion and migration, mitigating chemotherapy-induced damage, and promoting angiogenesis (Liu et al. 2022; Schuppan and Afdhal 2008). BM invasion is pivotal for tumor metastasis (Chang and Chaudhuri 2019), spotlighting the significance of BM-related genes (BMGs) in cancer research. Studies have shown that models based on BMGs effectively predict outcomes and immunotherapy impacts in solid tumors like lung adenocarcinoma (Zhang et al. 2023), pancreatic cancer (Zhou et al. 2024), and prostate cancer (Xie et al. 2024). In 2023, Sun W et al. (Sun et al. 2023) developed a prognostic model for HCC using BMGs, aiding clinicians in prognosis assessment and guiding immunotherapy and medication choices. The study affirmed the critical role of BM-related genes in HCC’s development and progression.

Long non-coding RNAs (lncRNAs) regulate mRNA, influencing disease processes and modulating cell function and gene expression, despite not encoding proteins directly (Rinn and Chang 2020). Research shows that lncRNAs’ abnormal expression can drive continuous tumor proliferation, metabolic anomalies, and metastasis (Zhang et al. 2021; Lin et al. 2021; Liu et al. 2021). Given the limited knowledge of BM-related lncRNAs, we aim to develop a new risk prediction model to enhance HCC’s clinical treatment and management. The study began by identifying HCC-associated BM genes through the Gene Expression Omnibus (GEO) and The Cancer Genome Atlas (TCGA) databases, then established and validated a risk model for HCC prognosis using TCGA’s LIHC sample data. Additionally, the study analyzed differences in immune cell presence, mutation rates, GSEA pathways, and drug sensitivity between high-risk and low-risk groups. Furthermore, this study validated the expression levels of six lncRNAs (GSEC, MIR4435-2HG, AC092614.1, AC127521.1, LINC02580, and AC008050.1) used to construct the model. Moreover, considering AC092614.1’s novel identification and unclear role in HCC, this study precisely located its presence in HCC cells using RNA fluorescence in situ hybridization (RNA FISH). Various assays, including CCK-8 and EdU proliferation, cell migration, wound healing, and Western Blot (WB) analysis, were employed to evaluate AC092614.1’s effect on HCC cell behaviors. These experimental approaches are intended to provide an experimental theoretical basis for AC092614.1 to be a possible prognostic marker and molecularly targeted therapy for HCC.

## Materials and Methods

### Data Collection and Processing

The gene expression profiles for HCC patients were obtained from the official websites of GEO (https://www.ncbi.nlm.nih.gov) (visited on June 29, 2022) and TCGA (https://portal.gdc.cancer.gov) (visited on June 29, 2022), with clinical data downloaded from TCGA’s official site (visited on June 29, 2022). Strawberry Perl (version 5.32.0.1, https://www.perl.org) and R software (version 4.1.1, https://www.r-project.org) were utilized for data integration and gene ID conversion, yielding gene expression matrices and clinical data files. This study downloaded 222 BMGs from the BM-BASE database (https://bmbase.manchester.ac.uk) (accessed on June 29, 2022). The differential expression analysis of BMGs was conducted using the “DESeq2” package in the R software (Version 4.1.1, https://www.r-project.org). Genes meeting the criteria of a P value < 0.05 and |logFC| ≥ 1.0 were considered as differentially expressed genes (DEGs) (Supplementary Table S1). Differentially expressed BMGs in HCC were obtained by intersecting DEGs with 222 BMGs. The protein interaction network of BMGs was constructed and visualized using STRING (https://cn.string-db.org) and Cytoscape software (version 3.9.1, https://cytoscape.org). The “limma” package in R software was utilized to select BM-related lncRNAs based on a Pearson correlation coefficient (r) > 0.50 and *P* < 0.001 (Supplementary Table S2).

### Construction of the BM-Related lncRNA Prognostic Model

The study constituted a cohort of 370 HCC patients with comprehensive clinical data from the TCGA database, which was randomly divided into training and validation sets in a 1:1 proportion. Univariate Cox regression was used to select lncRNAs closely associated with HCC survival, followed by further selection through the LASSO (least absolute shrinkage and selection operator) regression equation. A BM-related lncRNA prognostic model was then constructed using the “glmnet” package in R software. The formula for risk score was as follows: RISK SCORE= $\sum _{{{\text{i}} = 1}}^{{\text{n}}} {\text{Coefi}}\,\times\,{\text{Expi}}$. Where “Coef” represents the coefficient of lncRNAs associated with survival, and “Exp” is the expression level of each retained lncRNA. Based on the median value of the RISK SCORE, subgroups of high-risk and low-risk HCC patients were established in the TCGA cohort (Supplementary Table S3). The difference in survival between the low and high risk groups was analysed by Kaplan-Meier curves using the ‘survival’ and ‘survminer’ packages in the R software.

### Construction and Verification of the Predictive Model

Univariate and multivariate Cox regression analyses were performed to evaluate whether RISK SCORE serves as an independent prognostic factor for HCC patient survival. The performance of the model was evaluated using ROC curves, DCA curves, and calibration curves. Patients with clinical characteristics labeled as “unknown” were excluded in this step.

### Immunological Function Scoring and Tumor Mutation Burden Analysis

The TIMER, CIBERSORT, CIBERSORT-ABS, QUANTISEQ, MCPCOUNTER, XCELL and EPIC algorithms were applied to estimate the abundance of immune cells in high and low risk groups. Immune cell assessment using the single-sample gene set enrichment analysis (ssGSEA) algorithm using the “GSVA” package in R software (Hänzelmann et al. 2013). In addition, we compared the expression levels of gene markers related to immune checkpoints between the 2 risk cohorts for the potential value of the predictive model in immunotherapy. Regarding TP53 mutation status, this study utilized Kaplan-Meier curves to analyze the survival outcomes between high and low-risk subgroups under different TP53 mutation conditions. Total mutation burden (TMB) was calculated by defining the total number of mutations per megabyte of tumor tissue, with the intermediate TMB score used as a threshold to categorize patients into high TMB and low TMB groups. A comprehensive prognostic analysis of HCC patients was performed by integrating RISK SCORE.

### Functional Enrichment and Drug Sensitivity Analysis

In the pathways of the high- and low-risk groups, the enrichment score was calculated by GSEA using the “clusterprofiler” and “enrichplot” packages in R software, where *p* < 0.05 considered statistically significant. Five of the most important pathways were identified by Gene Ontology (GO) and Kyoto Encyclopedia of Genes and Genomes (KEGG). Eventually, the drug sensitivity analysis between different groups relied on the ‘oncoPredict’ package in R software.

### Experimental Materials and Cell Cultures

The human normal hepatocyte cell line WRL68 and HCC cell lines SMMC-7721, SK-HEP-1, LM3, HUH-7 and MHCC-97 H were donated to the Biliary and Pancreatic Surgery Laboratory of Tongji Hospital, Huazhong University of Science and Technology, China. DMEM (high glucose) and 10% foetal bovine serum were purchased from Gibco, USA; the 0.25% trypsin digestion solution was purchased from Wuhan Ltd. FISH probes and kits were purchased from Guangzhou Ribo Biotechnology Co. Transwells and Matrigel matrix gel were purchased from Corning, USA; rabbit anti-LAMB1 antibody, rabbit anti-collagen IV antibody, rabbit anti-human vimentin antibody, mouse anti-E-cadherin antibody, rabbit anti-human cell cycle protein E1 antibody, rabbit anti-human cyclin-dependent kinase 2 (CDK2) antibody, rabbit anti-human anti-Kip1 (P27) antibody, rabbit anti-human GAPDH antibody, and horseradish peroxidase-labelled sheep anti-mouse and anti-rabbit IgG (secondary antibody) were all purchased from Wuhan Sanying Biotechnology Co Ltd. RIPA lysis buffer and an ultrasensitive ECL chemiluminescent ready-to-use substrate kit were purchased from Wuhan PhD Bioengineering Co. Ltd. PVDF membranes were purchased from Sigma⁃Aldrich, USA. Small interfering RNA for AC092614.1 was purchased from Shanghai Bioengineering Co Ltd. Cells were cultured in high glucose DMEM with foetal bovine serum at 37 °C in a 5% CO2 incubator.

### Quantitative Real-Time PCR (qRT-PCR)

The expression levels of six lncRNAs were measured by qRT-PCR. Total RNA was extracted with the TRIzol. Reverse transcription was performed using the PrimeScriptTM RT Reagent Kit, and qRT-PCR analysis was performed using the SYBR® Premix Ex TaqTM II Kit. The thermal cycle settings are as follows: ①95 °C 30s, ② (95 °C 5s, 60 °C 30s) × 40 cycles. ③95 °C 15s, ④ (60 °C 60s, 95 °C 15s) × 1 cycle. GAPDH was selected as an internal reference, and the 2^−ΔΔCt^ method was used to calculate the relative expression levels of target genes. The Sequence of gene-specific primers used for qRT-PCR is shown in Supplementary Table S4.

### Cell Transfection and Grouping

All cell lines were cultured in complete DMEM medium, which contained 10% fetal bovine serum and 1% penicillin-streptomycin in DMEM medium. Optimal growth conditions for HUH-7 and MHCC-97 H cell lines were selected, and the cells were seeded in 6-well plates at a seeding density of 2 × 10^5^ cells per well. When cells had attached to the wells and reached 50–60% confluence, they were transfected with siRNA (either siNC or si-AC092614.1#3), and after 6 h, the medium was removed and fresh DMEM with supplements was added. The above steps were performed in strict accordance with Shanghai Bioengineering’s “Instructions for use of miRNA products”. Stably expressing cell lines were screened, and transfection efficiency was tested by qRT-PCR.

### RNA FISH

To determine the subcellular localization of AC092614.1 in HCC cells, we conducted an analysis of AC092614.1 in HUH-7 and MHCC-97 H cell lines using RNA FISH. The prepared cells were digested, neutralized and counted according to the RNA-FISH Product Instructions for Use from Ribo Bio Ltd. before the experiment and hepatoma cells were cultured. After washing with permeabilizing solution, SuperHybS solution containing the AC092614.1 RNA probe was added to the samples, after which the samples were observed, and images were captured using a laser confocal fluorescence microscope. Quantitative analysis was performed using ImageJ software (V1.8.0).

### CCK-8 Cell Proliferation Assay

The proliferation of HCC cells was assessed using CCK-8. The transfected hepatoma cells were detached, neutralized, counted, and plated in 96-well plates at a density of approximately 3 × 103 cells, with 5 replicate wells set up for each group and 4 time points (6 h, 24 h, 48 h, 72 h and 96 h) used for testing. The treated 96-well plates were placed in a cell culture incubator. After the cells were cultured for 6 h, 24 h, 48 h, 72 h and 96 h, the medium was discarded, and colour development solution was added to the wells and incubated for 2 h in a constant temperature incubator protected from light. The OD value at 450 nm was measured using an enzyme marker, and the growth curve was plotted. Each group of experiments was repeated 3 times.

### EdU

The proliferation of HCC cells was evaluated using Edu. EdU immunolabelling was performed using the Click-iT EdU-555 Cell Proliferation Assay Kit (Servicebio, G1602). hepatoma cells were labelled, fixed and permeabilised using EdU. After click reaction, nuclear staining of cells was performed. Analysis was performed using an inverted fluorescence microscope.

### Transwell Assay

The invasion capability of HCC cells was determined using Transwell. HCC cells selected for adherence to log phase were starved in serum-free medium for 12–24 h, then washed, digested, neutralised, centred and resuspended in serum-free medium. In the invasion group, 2 × 105 HCC cells were grown in the Transwell chamber; in the migration group, 1 × 105 HCC cells were grown in the Transwell chamber. The lower chamber was filled with 600 µl of DMEM containing 20% foetal bovine serum, and the cells were placed in a cell culture incubator for 24–48 h. The culture medium was removed from the upper and lower chambers of the Transwell chamber, which were then washed, fixed, stained, washed again, and dried in an oven. The number of cells on the surface of the lower chamber of the Transwell was then observed under an orthostatic microscope, and five fields of view were randomly selected for photographic counting.

### Wound-Healing Assay

The migratory capacity of HCC cells was assessed through a wound healing assay. Choose HCC cells with favorable growth conditions and plate them in a 6-well plate. After cell attachment, make three parallel and evenly spaced scratches in each well using the tip of a pipette, substituting the culture medium with serum-free culture medium.

### Protein Extraction and WB

The expression of relevant proteins was detected using WB. After digestion of each group of cells, the lysis buffer was added, and after sonication and centrifugation, 4 µl of protein was collected for assessment. Loading buffer (5×) was added to each tube of protein supernatant in the corresponding proportion, followed by boiling at 95 °C for 10 min. After total protein extraction, SDS‒PAGE gels were prepared, after which protein loading, electrophoresis, membrane transfer and incubation with antibody were performed. The antibodies included: LAMB1, collagen IV, laminin, E-cadherin, E1, CDK2, P27, and GAPDH. After secondary antibody incubation and band washing, an ECL chemiluminescence solution was prepared and incubated with the target bands. Gel imager was used to capture images, and quantitative analysis was performed using ImageJ software (V1.8.0).

### Statistical Analysis

Continuous variables were presented as mean ± standard deviation (mean ± SD). Two-group comparisons of continuous variables were conducted using the *t* test for variables conforming to a normal distribution, while the Mann–Whitney *U* test was used for variables with a non-normal distribution. Count data were analyzed using the chi-square test. Univariate and multivariate survival analyses were carried out using Cox proportional hazards regression models. Kaplan-Meier survival analyses were performed, and the log-rank test was used for comparison. Statistical analyses were performed using R statistical software and Strawberry Perl software. A significance level of *p* < 0.05 was considered statistically significant.

## Results

### Selection of Differentially Expressed BM-Related lncRNAs

**Fig. 1:**
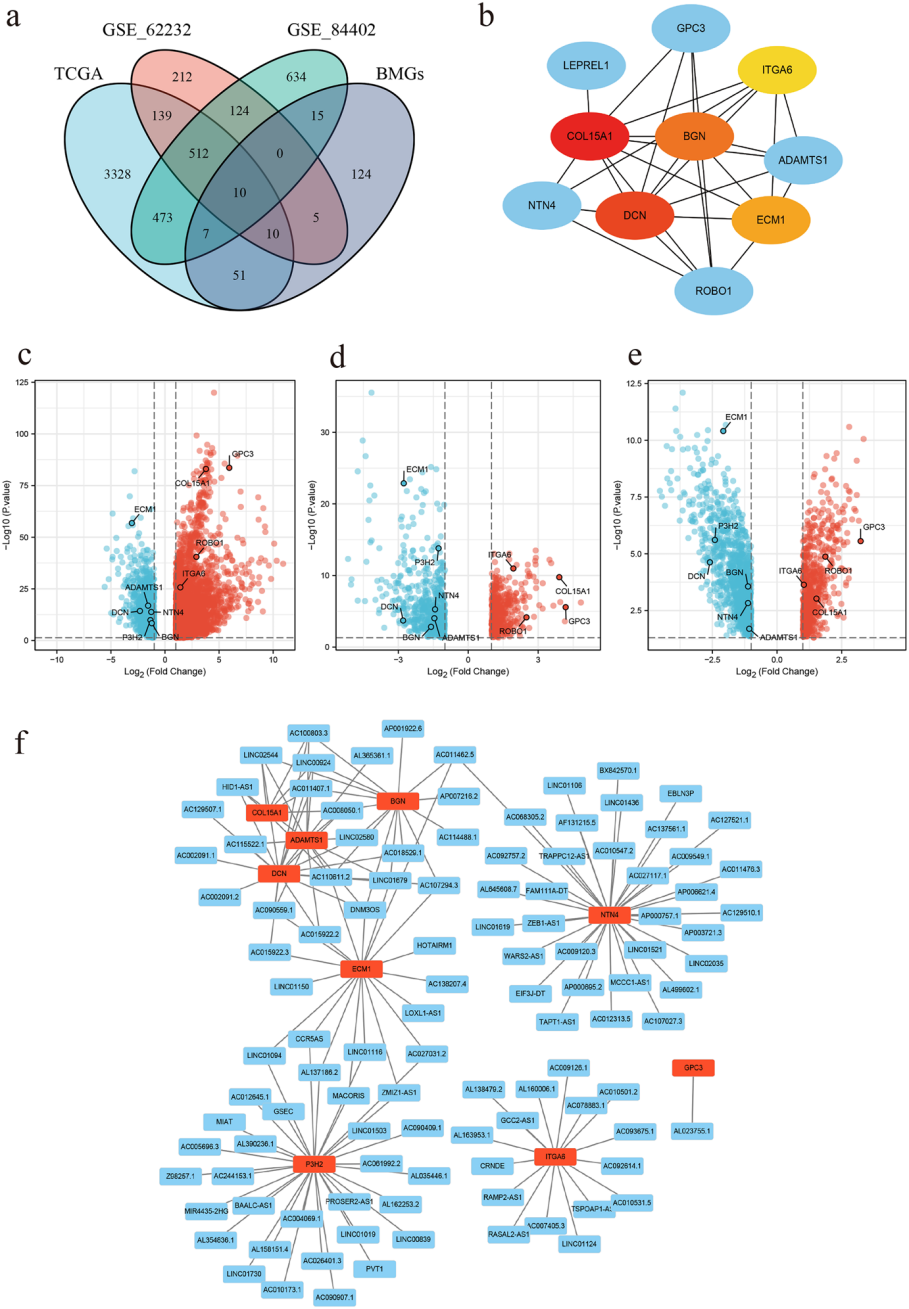
Screening of differentially expressed BMGs in HCC **a** Venn diagram of HCC-related differentially expressed genes and BMGs from three databases. **b** Protein interactions of screened BMGs. **c**–**e** The expression of screened BMGs in the TCGA-LIHC, GEO_84402 and GEO_62232 datasets. **f** Network connectivity diagram showing correlation between BMGs and lncRNAs

Using the criteria of *P* < 0.05 and and |logFC|≥1.0, this study selected 4530, 1175, and 1012 differentially expressed genes from the TCGA-LIHC, GEO_84402, and GEO_62232 datasets, respectively, with 522 genes showing significant differences in all three datasets. 222 disease-related BMGs were downloaded from the BM-BASE database. The intersection of BMGs and differentially expressed genes in HCC resulted in 10 differentially expressed BMGs in HCC (Fig. 1a). Protein-protein interactions were found among these BMGs (Fig. 1b), including 6 downregulated genes and 4 upregulated genes (Fig. 1c–e). Based on the selected BMGs, 110 BM-related lncRNAs were screened in the TCGA database with the criteria of Pearson correlation coefficient (r) > 0.5 and *P* < 0.001, except for GPC3, which showed no significant correlation with lncRNAs (Fig. 1f).

### Prognostic Model Construction Based on BM-Related lncRNAs

The TCGA dataset was divided into training (*n* = 185) and testing (*n* = 185) sets from the entire cohort of 370 patients. Univariate Cox regression analysis was performed to evaluate the prognostic value of BM-related lncRNAs in HCC patients, and 8 BM-related lncRNAs significantly associated with survival were identified using a significance level of *P* < 0.05 (Supplementary Fig. 1a). Further analysis using LASSO regression was conducted to calculate the optimal penalty coefficient and ultimately select 6 BM-related lncRNAs to construct a risk prediction model (Supplementary Fig. 1b, c). The risk score of the model is as follows: RISK SCORE = 0.5073*GSEC + 1.6375*MIR4435-2HG + 1.4531*AC092614.1–2.0624*AC127521.1–1.7930*LINC02580–1.0715*AC008050.1. Furthermore, to clarify the correlation between individual genes in the model and BMGs, a correlation heatmap was used to display the relationship between the 6 model genes and BMGs (Supplementary Fig. 1d), with GPC3 showing no significant correlation with lncRNAs. A circle plot was used to visualize the correlations between the 6 lncRNAs (Supplementary Fig. 1e).

### Validation of the BM-Related lncRNA Prognostic Model

Based on a median risk score of 1.05, the TCGA cohort was divided into high-risk and low-risk groups. The K-M survival curve indicates that the prognosis for HCC patients with high risk scores is significantly worse than for those with low risk scores (Supplementary Fig. 2a). Compared to the low-risk group, the mortality rate in the high-risk group increases over time (Supplementary Fig. 2b). Univariate and multivariate Cox regression analyses both indicate that RISK SCORE and stage independently predict HCC survival rates (*P* < 0.01, Supplementary Fig. 2c, d).

The performance of the prognostic risk model was assessed through ROC curves, DCA curves, and calibration curves. In the entire cohort, the model’s predicted Area Under Curve (AUC) for 1, 3, and 5 years are 0.757, 0.726, and 0.631, respectively; for the training set, the 1, 3, and 5 years AUC are 0.773, 0.753, and 0.690, respectively; for the test set, the 1, 3, and 5 years AUC are 0.754, 0.717, and 0.585, respectively (Supplementary Fig. 3a). Multivariate time-dependent ROC curves show that the risk score has the strongest predictive power (Supplementary Fig. 3b). Calibration curves reveal the actual survival outcomes of HCC patients at 1, 3, and 5 years closely align with predictions (Supplementary Fig. 3c). DCA curves indicate the model’s superior accuracy (Supplementary Fig. 3d).

### Survival Analysis

Stratification survival analysis reveals that, upon stratification by pathological grades (I–II vs. III-IV) and age groups (< 65 vs. >=65 years), there are significant survival differences between the high and low-risk groups. Upon gender stratification, significant survival differences between high and low-risk groups were evident among male patients, while no significant differences were noted among female patients (Supplementary Fig. 4).

### Analysis of the Immune Microenvironment

To delve deeper into the correlation between risk scores and immune cells, this research employed seven algorithms to depict immune cell infiltration heatmaps, revealing significant disparities in the immune microenvironments of high and low-risk groups (Supplementary Fig. 5a). ssGSEA analysis indicated that, compared to the high-risk group, pathways such as APC, CCR, Cytolytic activity, HLA, para inflammation, TYP_INF-I, and INF-II were markedly activated in the low-risk group (Supplementary Fig. 5b). Following this, differential analysis of immune checkpoints revealed that most immune checkpoint proteins exhibited higher expression in the high-risk group than in the low-risk population (Supplementary Fig. 5c).

### Analysis of Tumour Mutations

An analysis of mutation data for both high and low-risk groups indicated a higher frequency of gene mutations within the high-risk group compared to the low-risk group (Supplementary Fig. 6a, b). A further review of TP53 mutation prevalence across the cohort revealed TP53 mutations in 35% of the high-risk group versus 16% of the low-risk group (Supplementary Fig. 6c), demonstrating a significant correlation between risk scores and TP53 mutation frequencies. Comprehensive analysis between TP53 status and risk scores shows that, regardless of TP53 being wild-type or mutant, patients in the high-risk group exhibit worse prognoses than those in the low-risk group. Additionally, Kaplan–Meier curves for patients with either TP53 mutation or wild-type in both high and low-risk groups show overlap, suggesting TP53’s limited efficacy in stratifying prognoses, in contrast to the superior performance of BM-related lncRNA models (Supplementary Fig. 6d). Comparison of TMB scores between high and low-risk groups, based on TCGA somatic mutation data, reveals significantly higher TMB scores in the high-risk group compared to the low-risk group (Supplementary Fig. 6e). Furthermore, Kaplan–Meier curves demonstrate that BM-related lncRNA models surpass TMB scores in prognostic accuracy for patient survival (Supplementary Fig. 6f, g).

### GSEA Enrichment and Drug Sensitivity Analyses

**Fig. 2:**
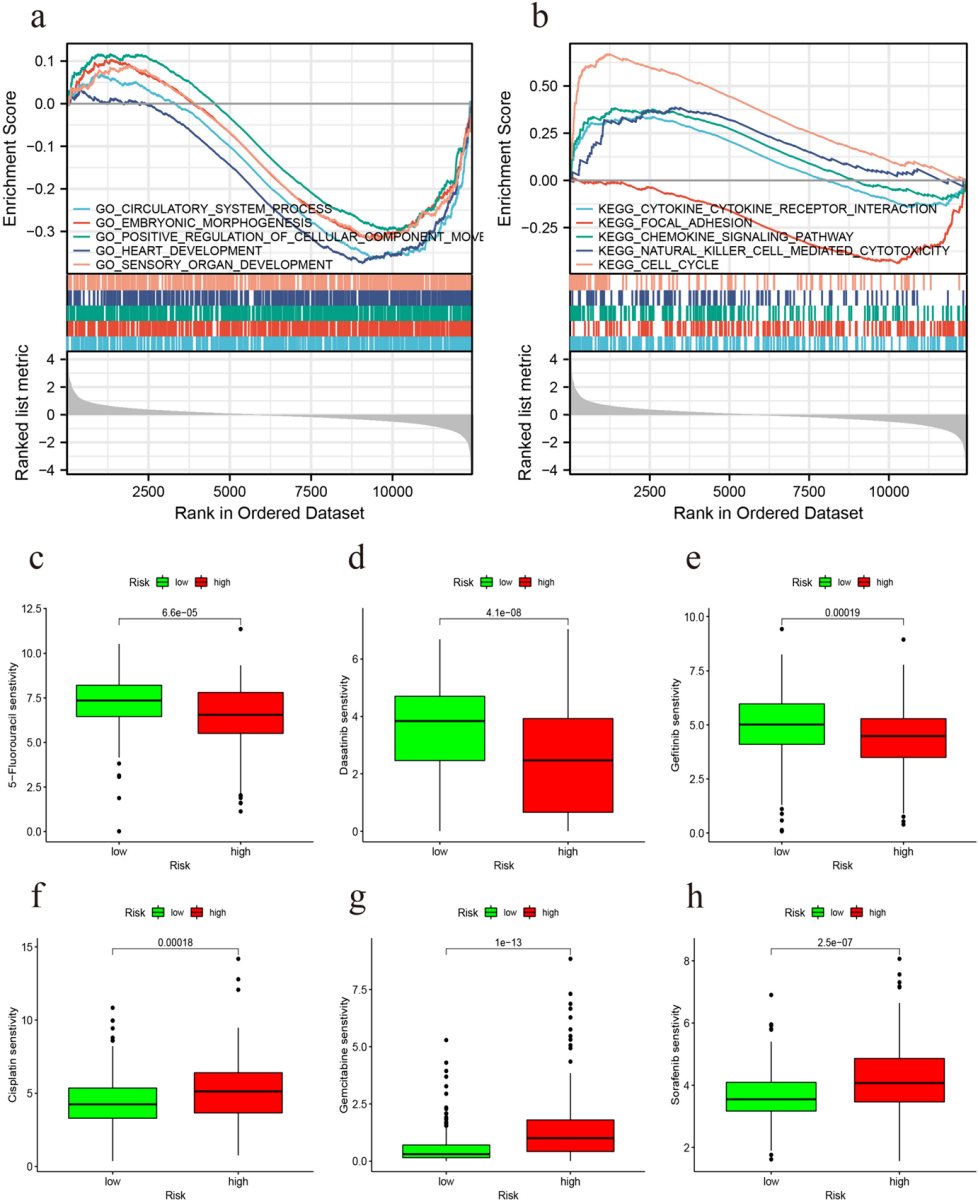
GSEA enrichment analysis and drug sensitivity analysis **a**, **b** GSEA enrichment analysis for high and low risk groups. **c**–**h** Drug sensitivity analysis between high and low risk subgroups of patients

GSEA enrichment and drug sensitivity analyses were performed for both high and low-risk groups. GO enrichment analysis reveals the low-risk group is associated with various changes, such as processes in the circulatory system, embryonic morphogenesis, positive regulation of cellular component movement, and development of the heart and sensory organs. KEGG enrichment analysis revealed that high-risk groups are linked to processes including cytokine receptor interactions, chemokine signaling pathways, natural killer cell-mediated cytotoxicity, and cell cycle pathways, whereas low-risk groups are associated with focal adhesion pathways (Fig. 2a, b). Drug sensitivity analysis indicated that the high-risk group exhibits greater sensitivity to 5-fluorouracil, dasatinib, and gefitinib, whereas the low-risk group shows higher sensitivity to cisplatin, gemcitabine, and sorafenib (Fig. 2c–h).

### Detection of lncRNAs Expression Levels in Prognostic Risk Model

**Fig. 3:**
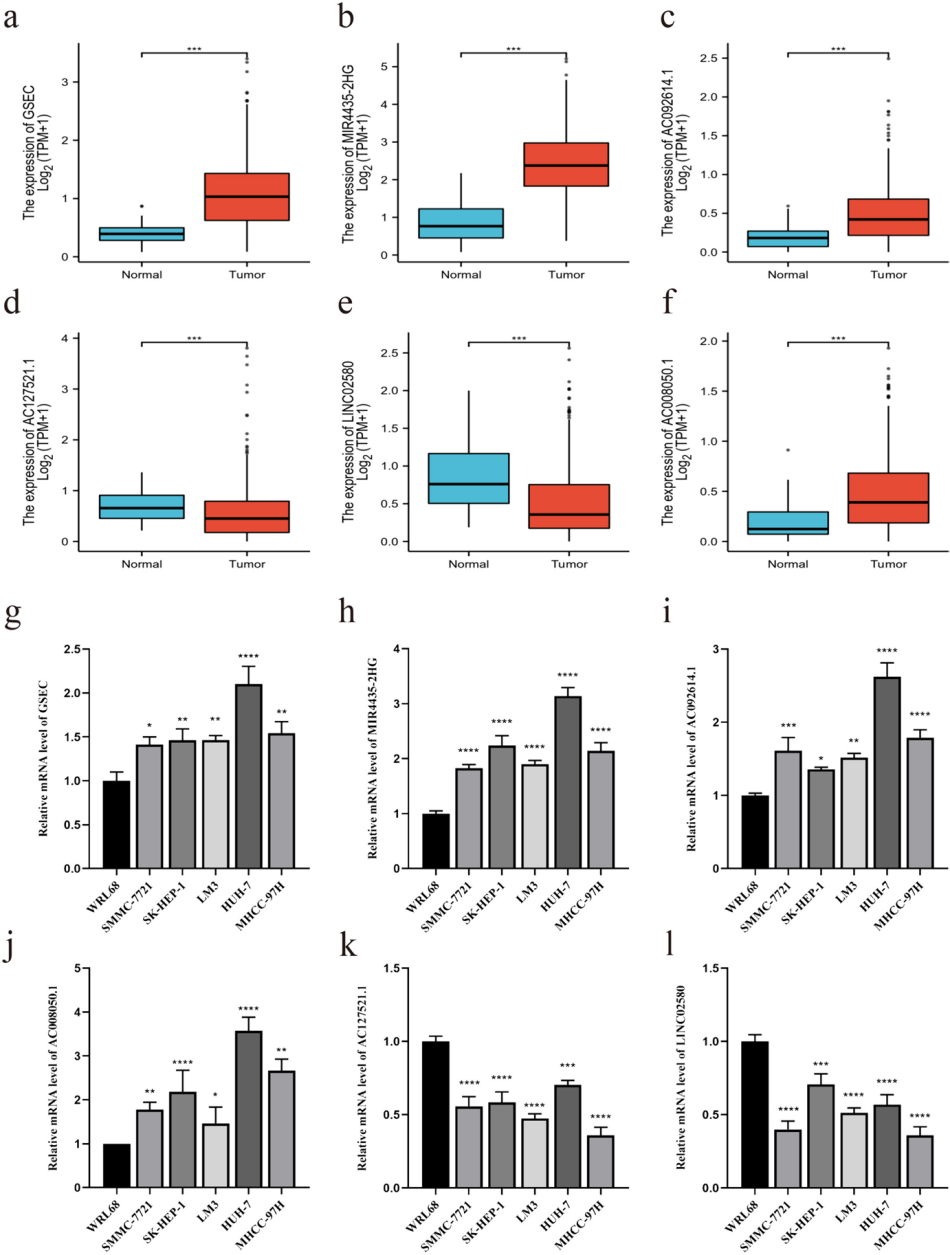
Detection of expression levels of six lncRNAs based on TCGA data **a**–**f** Expression of GSEC, MIR4435-2HG, AC092614.1, AC008050.1, AC127521.1 and LINC02580 in HCC tissues and normal tissues. **g**–**l** Expression of GSEC, MIR4435-2HG, AC092614.1, AC008050.1, AC127521.1 and LINC02580 in normal hepatocytes and hepatoma cells. ∗*P* < 0.05, ∗∗*P* < 0.01, ∗∗∗*P* < 0.001, ∗∗∗∗*P* < 0.0001

Within the TCGA dataset, the expression levels of six lncRNAs, utilized for model construction, were compared between HCC tissues and normal liver tissues. Results indicated that GSEC, MIR4435-2HG, AC092614.1, and AC008050.1 exhibited higher expression levels in HCC tissues compared to normal liver tissues, whereas AC127521.1 and LINC02580 showed lower expression levels in HCC tissues than in normal liver tissues (Fig. 3a–f). qRT-PCR assays revealed analogous outcomes in WRL68 and HCC cell lines (Fig. 3g–l). Subsequently, this study selected AC092614.1 for further validation due to its relatively high expression and the fact that it has never been validated in HCC before.

### Localization of AC092614.1 and Impact on the Proliferative Capacity of Hepatoma Cells

**Fig. 4:**
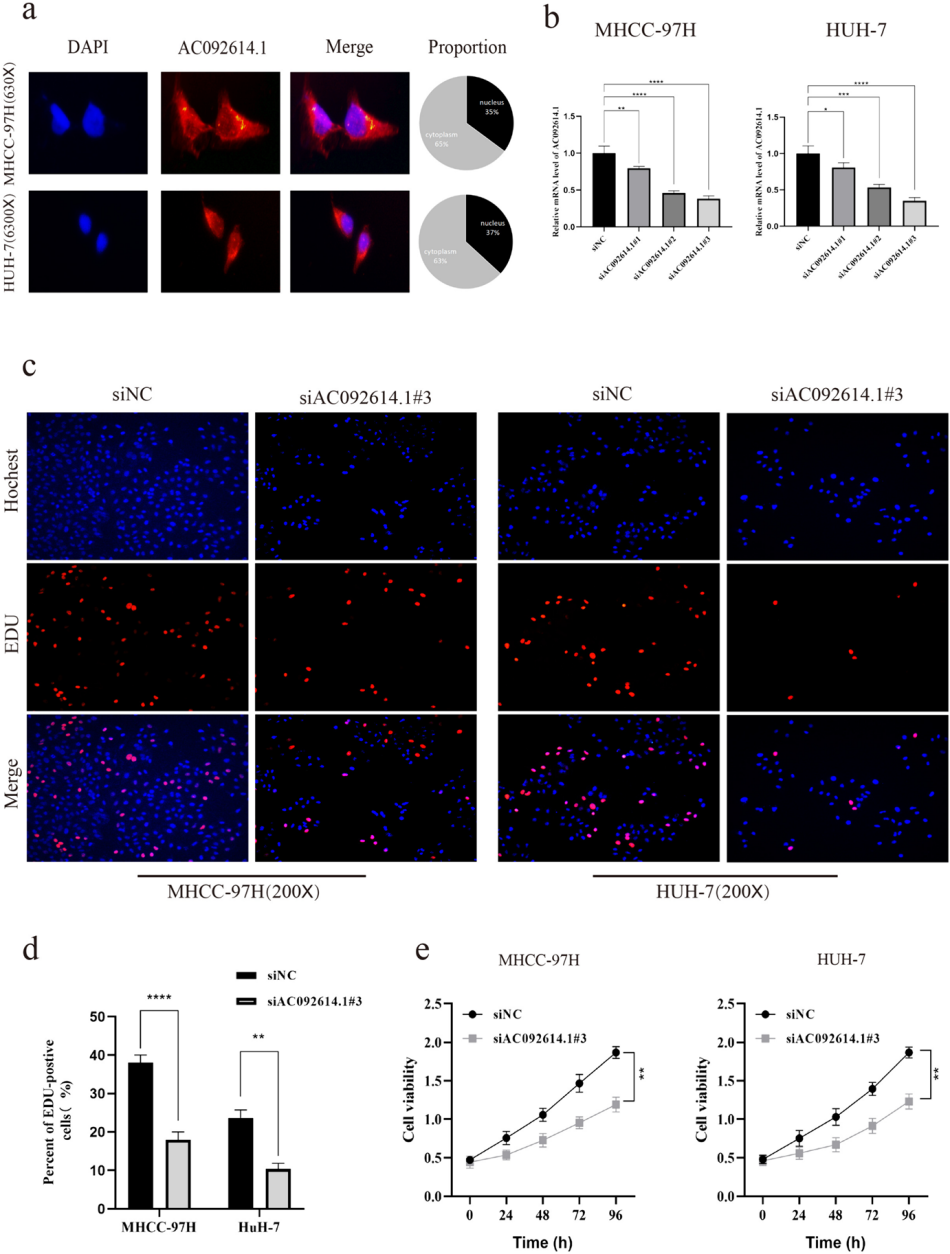
Validation of AC092614.1 localization and function in hepatoma cells **a** Localization of AC092614.1 in HCC cells determined by FISH. **b** qRT-PCR to verify the transfection efficiency of AC092614.1 down-expression. **c**–**e** CCK-8 and EdU assays to assess the effect of AC092614.1 on the proliferation of HCC cells. ∗*P* < 0.05, ∗∗*P* < 0.01, ∗∗∗*P* < 0.001, ****P < 0.0001

FISH analysis revealed higher levels of AC092614.1 in the cytoplasm than in the nucleus, suggesting that AC092614.1 may function in the cytoplasm (Fig. 4a). Furthermore, siRNA was utilized to decrease AC092614.1 expression in HUH-7 and MHCC-97 H cell lines, validating transfection efficiency (Fig. 4b). CCK-8 and EdU assays demonstrated that diminished expression of AC092614.1 inhibits the proliferation of HUH-7 and MHCC-97 H cell lines (Fig. 4c–e).

### Effect of AC092614.1 on the Invasion and Migration Ability of Hepatoma Cells

**Fig. 5:**
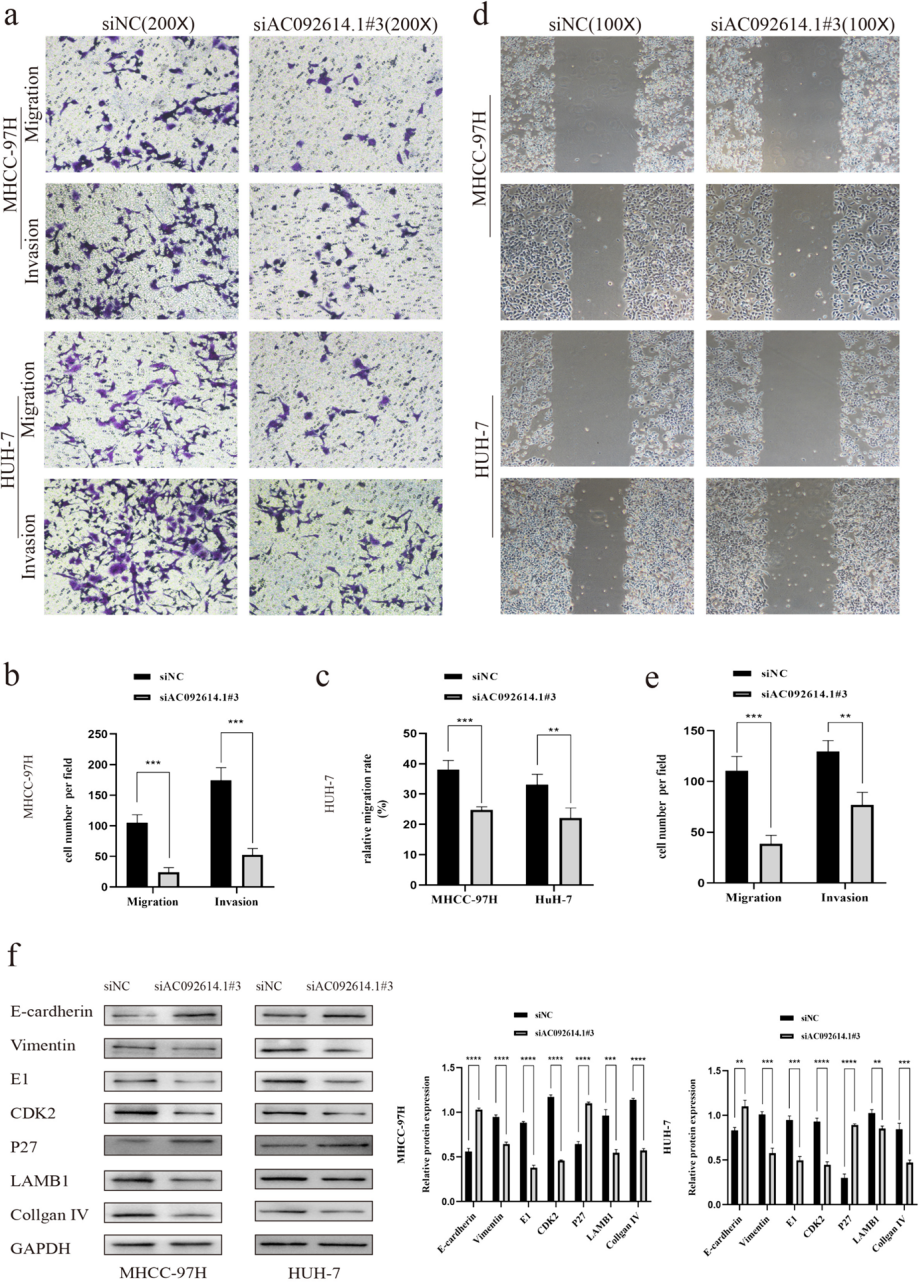
Validation of AC092614.1 function in hepatoma cells **a**–**c** Reducing the expression of AC092614.1 inhibited the invasive metastasis of HCC cells. **d**, **e** Reducing the expression of AC092614.1 inhibited HCC cells metastasis. **f** Reducing the expression of AC092614.1 inhibited the expression of basement membrane-associated component proteins, HCC cell cycle proteins and EMT-associated proteins. ∗*P* < 0.05, ∗∗*P* < 0.01, ∗∗∗*P* < 0.001, ∗∗∗∗*P* < 0.0001

Through Transwell and wound healing assays, this study investigated the impact of AC092614.1 on the invasive and migratory capacities of HCC cells. Results showed that lowering AC092614.1 expression inhibits the migration and invasion of HUH-7 and MHCC-97 H cell lines (Fig. 5a–e). WB analysis of the effect of reducing AC092614.1 expression on key BM component proteins, cell cycle-related proteins, and invasion and metastasis-related proteins in HCC cells showed that a decrease in AC092614.1 expression leads to reduced levels of Vimentin, E1, CDK2, LAMB1, and collagen IV, as well as increased levels of E-cadherin and P27 (Fig. 5f).

## Discussion

HCC exhibits marked heterogeneity, aggressive invasion, and a generally bleak prognosis (Chan et al. 2022). As mentioned previously, targeting BMGs or lncRNAs represents a potential cancer treatment strategy, particularly BM-related lncRNAs, which are likely to become ideal anticancer drug targets by regulating BM components and affecting tumor occurrence and development (Zhou et al. 2021).

Our study established a prognostic model for HCC risk based on BM-related lncRNAs. The model’s effectiveness was evaluated using ROC, DCA, and calibration curves, with AUC values of 0.757, 0.726, and 0.631 for 1-year, 3-year, and 5-year survival, indicating its reliability. Jin Z et al. (Jin et al. 2023) identified 10 BM and immune checkpoint-related lncRNAs through machine learning in the TCGA and constructed a prognostic model for HCC. The AUCs for 1-year, 3-year, and 5-year survival rates were 0.726, 0.697, and 0.675, respectively. Despite different lncRNAs in our model compared to Jin Z et al. both models similarly and effectively predict HCC prognosis, underscoring the models’ utility. Regarding immune infiltration, Jin Z’s findings on macrophages and regulatory T cells (Tregs) predominating in high-risk groups are mirrored by our CIBERSORT analysis in Supplementary Fig. 5A, highlighting BM-related lncRNAs’ crucial role in HCC’s immune infiltration. Prior studies link BM’s structure and function to tumor immunity. Laminins and collagen, the primary components of BM, where laminins can bind to specific receptors on nearly all immune cells, initiating signals that regulate immune cell function and migration (Simon and Bromberg 2017) while high collagen density can reduce the activity of cytotoxic T cells (Kuczek et al. 2019). Additionally, Matrix metalloproteinase-2-induced BM damage recruits macrophages, promoting tumor metastasis and immune suppression (Diwanji and Bergmann 2020). Our results affirm BM-associated lncRNAs’ significance to the immune microenvironment, where immune cell alterations in HCC closely link to prognosis and therapeutic outcomes (Yasuoka et al. 2020; Cao et al. 2020). In HCC, macrophages often display M2-like polarization, facilitating angiogenesis, tissue remodeling, and immune suppression (Argentiero et al. 2023; Wang et al. 2022a, b). Tregs mainly inhibit CD8 + T cell cytotoxicity and NK cell proliferation through cytokines like IL-4, IL-10, or TGF-β, intensifying immune suppression (Wang et al. 2012; Gao et al. 2007). Elevated Tregs and macrophages in high-risk groups signify poorer prognosis and stronger immune suppression, markedly influencing treatment outcomes. In conclusion, our research shows BM-related lncRNAs influence HCC prognosis through their impact on the immune microenvironment.

ssGSEA analysis revealed enhanced activation of immune response pathways in the low-risk group versus the high-risk group, critical for boosting immune cell function and ensuring vigilant tumor surveillance (Borden 2019; Zhou et al. 2023). Moreover, Additionally, higher expression of immune checkpoint proteins, such as CTLA4 and CD80, in the high-risk group indicates potential for improved outcomes through immunotherapy. TP53 mutations lead to increased tumor aggressiveness and worse prognosis (Wang et al. 2022a, b). With 35% of the high-risk group and 16% of the low-risk group showing TP53 mutations, it is speculated that the high mutation rate of TP53 in the high-risk group may be one of the reasons for the poor prognosis.

Chemotherapy is a common treatment for advanced HCC but the development of drug resistance presents a significant challenge to treatment and prognosis improvement, making the enhancement of chemosensitivity of great clinical importance (Chouhan et al. 2020; Villanueva et al. 2019; Bejjani and Finn 2022; Sadagopan and He 2024). Drug sensitivity analysis showed that low-risk patients respond better to cisplatin, gemcitabine, and sorafenib, while high-risk patients are more responsive to 5-fluorouracil, dasatinib, and gefitinib. Our model stratifies patients into distinct risk categories, offering a guide for selecting targeted treatments and personalizing therapy for HCC.

GSEC, MIR4435-2HG, AC092614.1, AC127521.1, LINC02580, and AC008050.1 were validated within HCC cell lines in this analysis. In colorectal cancer, GSEC influences tumor migration by inhibiting DHX36 function through G-quadruplex structures (Matsumura et al. 2017). MIR4435-2HG has been extensively studied for its ability to affect tumor development through multiple pathways, including the Wnt/β-catenin, DM2/p53, PI3K/AKT, and MAPK/ERK signaling pathway (Zhong et al. 2022). High expression of MIR4435-2HG has been proven to be associated with the pathological characteristics and poor prognosis of various tumors, such as colorectal and breast cancer (Yu et al. 2022; Ke et al. 2022; Ho et al. 2021). There is limited research on AC127521.1 and LINC02580, but studies have shown that AC127521.1 can promote the malignant biological behavior of acute myeloid leukemia by regulating the expression of SPNS3 mediated by MIR-139 (Hong et al. 2021). XU L’s research indicates that LINC02580 can regulate pathways related to EMT in HCC cells through specific binding to serine/arginine-rich splicing factor 1 (Xu et al. 2020). AC092614.1 and AC008050.1 were identified for the first time in this study, with results showing that high expression of AC092614.1 and low expression of AC008050.1 are associated with poor prognosis in HCC. AC092614.1, previously undiscovered and showing significant expression variability, was chosen for in vitro functional experimentation.

In this study, siRNA transfection was used to reduce the expression of AC092614.1 in HCC cells, and knocking down AC092614.1 expression weakened the proliferation, migration, epithelial-mesenchymal transition, and cell cycle progression of HCC cells. More importantly, reducing the expression of AC092614.1 led to a decrease in the expression of key BM component proteins LAMB1 and collagen IV in HCC cells, providing evidence of AC092614.1’s involvement in regulating the extracellular matrix of HCC cells.

This study faces limitations, notably in bioinformatics analysis, where it lacks robust external validation. The International Cancer Genome Consortium (ICGC) liver cancer database lacked sequencing results for GSEC, and the GEO_84402 and GEO_62232 databases involved in this study lacked complete clinical information of patients. Therefore, internal validation with the TCGA dataset and in vitro experimental validation were conducted in this study to compensate. The internal validation results of this study model show that the prognosis model constructed based on BM-related lncRNAs performs well, providing confidence for seeking available datasets for external validation in the future. Furthermore, this study has limitations in experimental validation. It did not perform histological level expression analysis of AC092614.1, lacks further in vivo experimental validation, and also lacks detection and validation of downstream molecules of AC092614.1, missing an exploration of the mechanistic links. Further, we aim to delineate AC092614.1’s mechanisms via RNA-seq, ChIP, and luciferase assays, consolidating in vitro findings through in vivo validations in HCC xenografts or transgenic mouse models. Additionally, for this predictive model, we will also conduct external validation in an independent patient cohort and demonstrate its clinical utility through prospective studies. In summary, the specific mechanisms by which AC092614.1 regulates the occurrence and development of HCC require further study, and this research provides fundamental theoretical and experimental support in this regard.

## Conclusions

In the presented study, the prognostic model based on BM-associated lncRNAs effectively predicts survival outcomes across different risk categories in HCC, highlighting its strong correlation with the immune microenvironment, immune checkpoints, mutation frequencies, and drug responsiveness. Significant differential expression of GSEC, MIR4435-2HG, AC092614.1, AC127521.1, LINC02580, and AC008050.1 was observed in HCC cell lines. Notably, AC092614.1 enhances HCC cell proliferation, invasion, and migration, positioning it as a novel prognostic indicator and a promising therapeutic target.

## Supplementary Information

Below is the link to the electronic supplementary material.

Supplementary material 1 (DOCX 4000.5 kb)

Supplementary material 2 (XLSX 189.2 kb)

## Data Availability

The datasets can be found in TCGA portal (https://portal.gdc.cancer.gov/), GEO portal (https://www.ncbi.nlm.nih.gov/geo/), and BM-BASE database (https://bmbase.manchester.ac.uk).
