# Supplementary material for: Identification and Validation of Basement Membrane Related LncRNA Signatures as a Novel Prognostic Model for Hepatocellular Carcinoma
Source: Biochem Genet. 2024 Apr 29;63(3):2100–19. doi: 10.1007/s10528-024-10797-3 (PMC12144066; doi:10.1007/s10528-024-10797-3)

**Supplementary Fig. 1** Construction of prognostic models for HCC patients based on BM-related lncRNAs (a) One-way COX analysis of BM-related lncRNAs. (b, c) LASSO regression to further screen lncRNAs and construct prognostic models. (d) Correlation analysis between BMGs and lncRNAs; (E) Correlation analysis between lncRNAs.


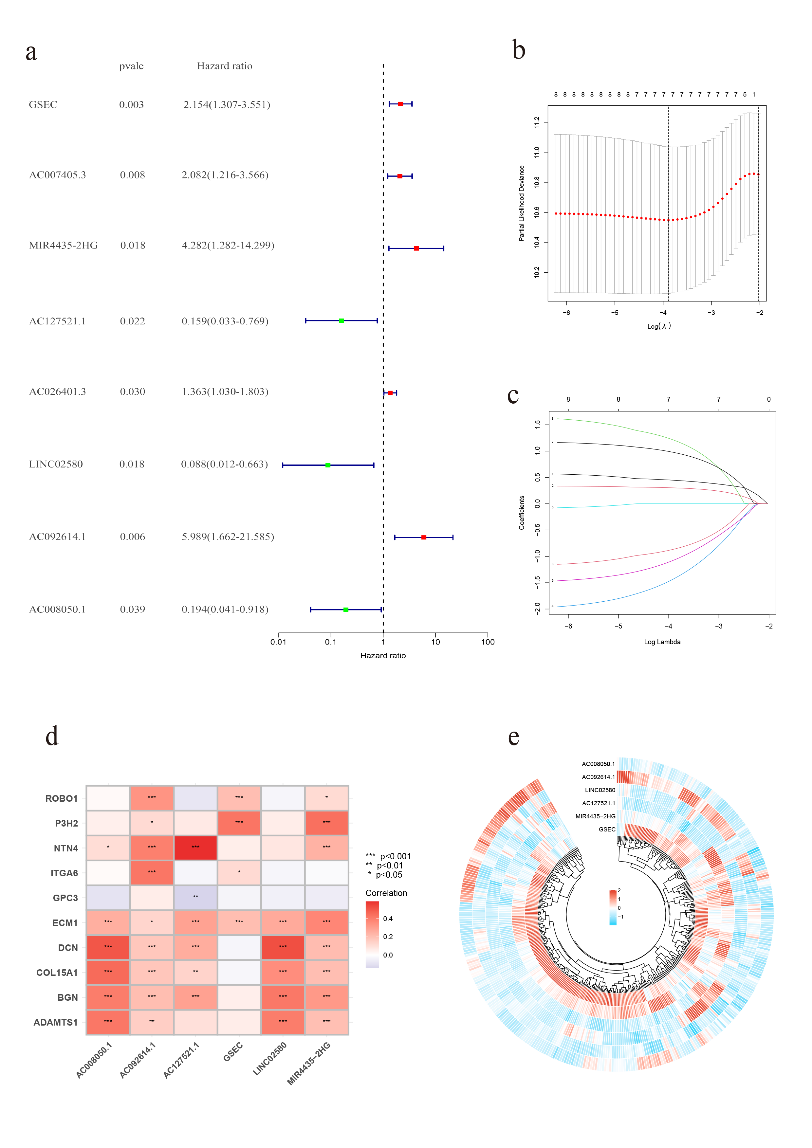


**Supplementary Fig. 2** Evaluation and validation of the prognostic risk model (a) K-M curves to evaluate the performance of the model. (b) scatter plot and heat map to validate the correlation between survival time, lncRNA and risk score. (c, d) One-factor and multi-factor Cox regression analysis (p<0.001).


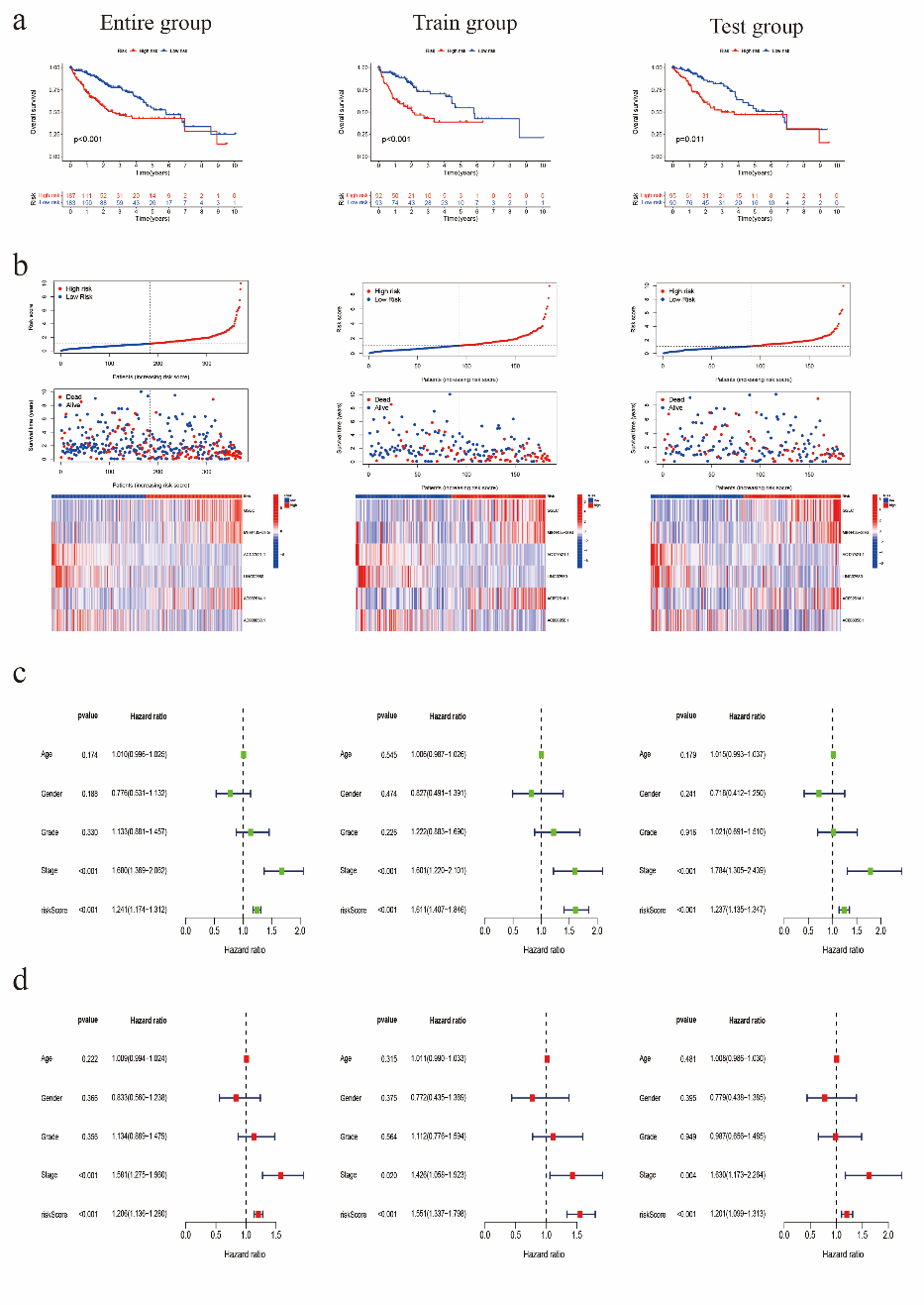


**Supplementary Fig. 3** Evaluation of prognostic risk models (a) ROC curves for predicting patients' overall survival at 1, 3 and 5 years. (b) Multivariate ROC curves for predicting patients' overall survival at 1 year. (c) Calibration curve analysis. (d) DCA curve analysis.


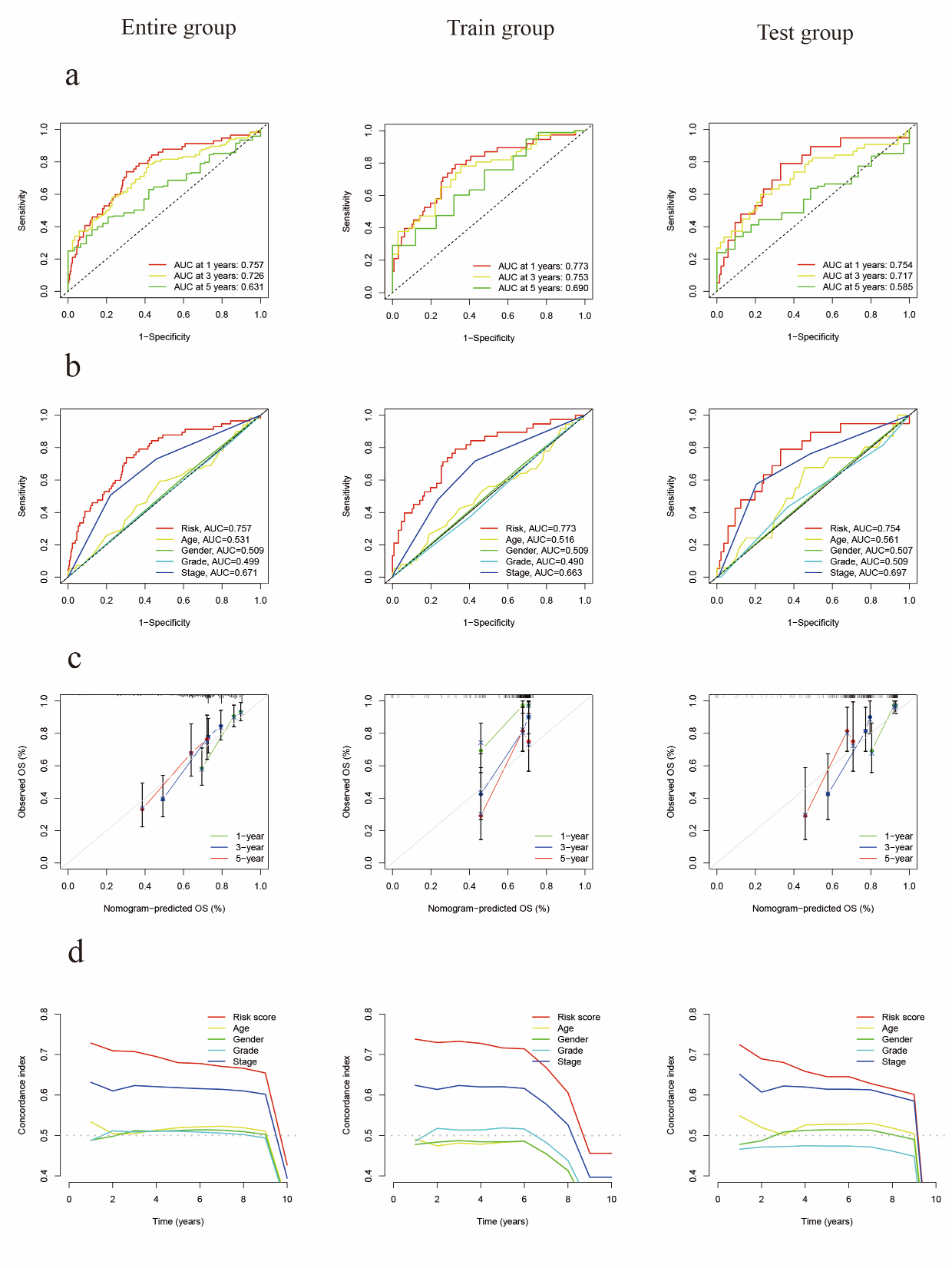


**Supplementary Fig. 4** Kaplan-Meier survival analysis (a-f) K-M curves showing survival differences between patients with HCC by pathological stage, age and sex.


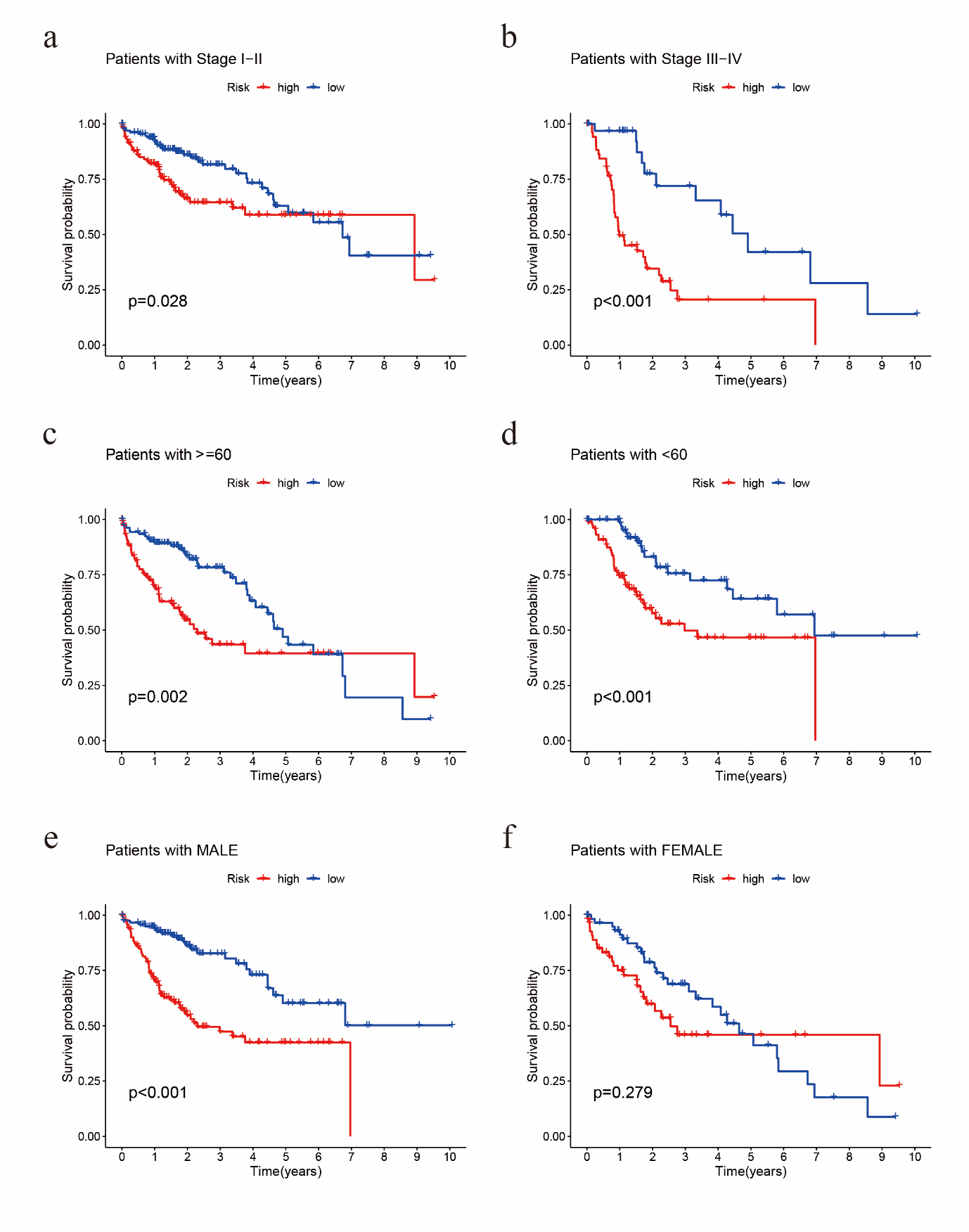


**Supplementary Fig. 5** Correlation assessment of tumour microenvironment risk scores (a) TIMER, CIBERSORT, CIBERSORT-abs, QUANTISEQ, MCP-counter, XCELL and EPIC algorithms were used to map immune cell infiltration heat in patients with different risk scores. (b) Immune function scores of high- and low-risk groups. (c) Differences in expression of immune checkpoint genes between high and low risk groups. *p<0.05, **p<0.01, ***p<0.001, ns not statistically different.


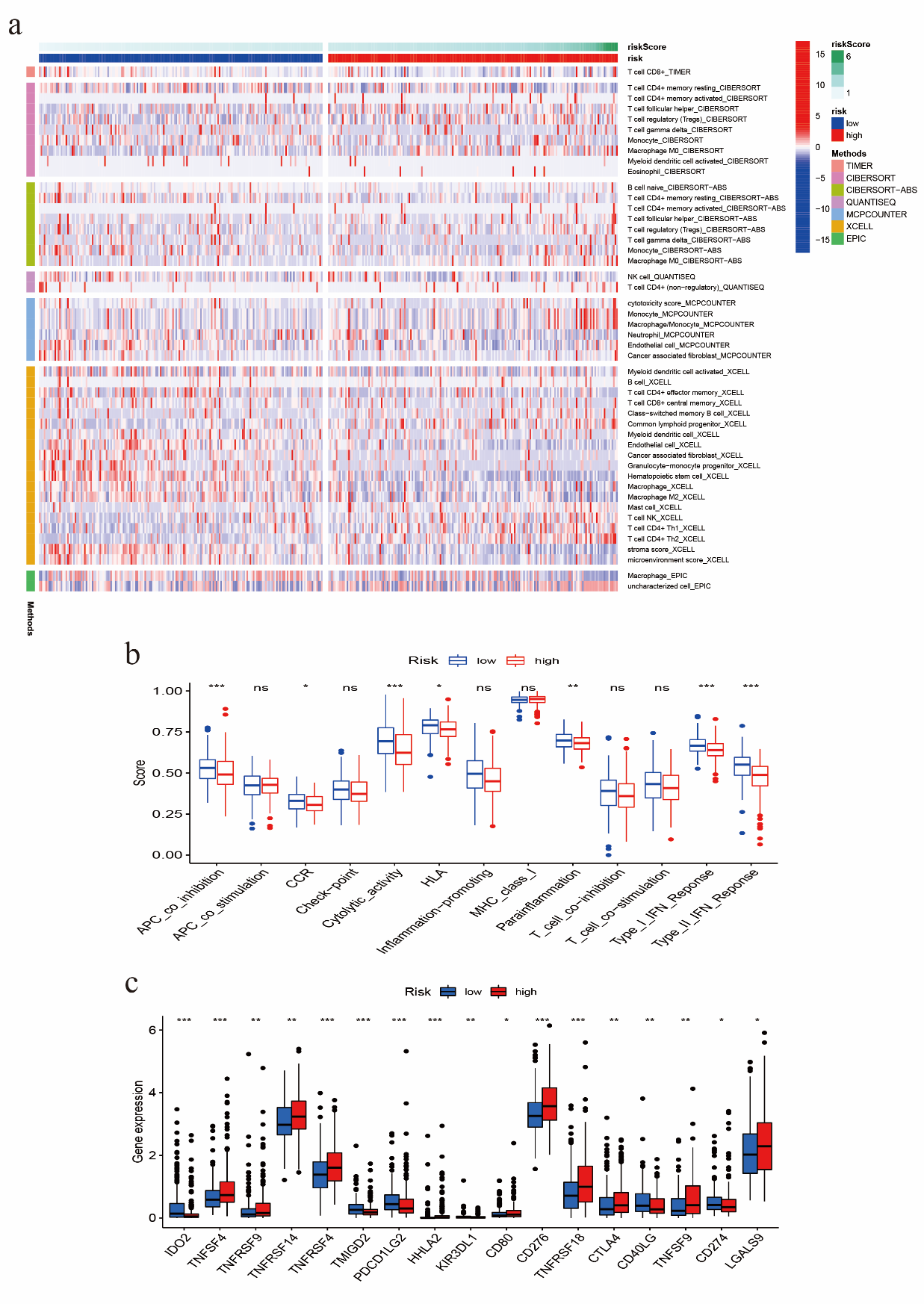


**Supplementary Fig. 6** Gene mutation and survival analysis between patients in high- and low-risk groups (a, b) Waterfall plots showing mutation information of high mutation frequency genes in the high-risk groups (a) and low-risk group (b). (c) Percentage of TP53 mutations in high- and low-risk subgroups. (d) Comparison of K-M curve analysis between high- and low-risk sugroups with different TP53 mutations. (e) Analysis of TMB differences between high- and low-risk groups. (f) Analysis of K-M curves in patients with different TMB levels. (g) Comparison of K-M curve analysis between high- and low-risk groups with different TMB. TMB: tumor mutational load.


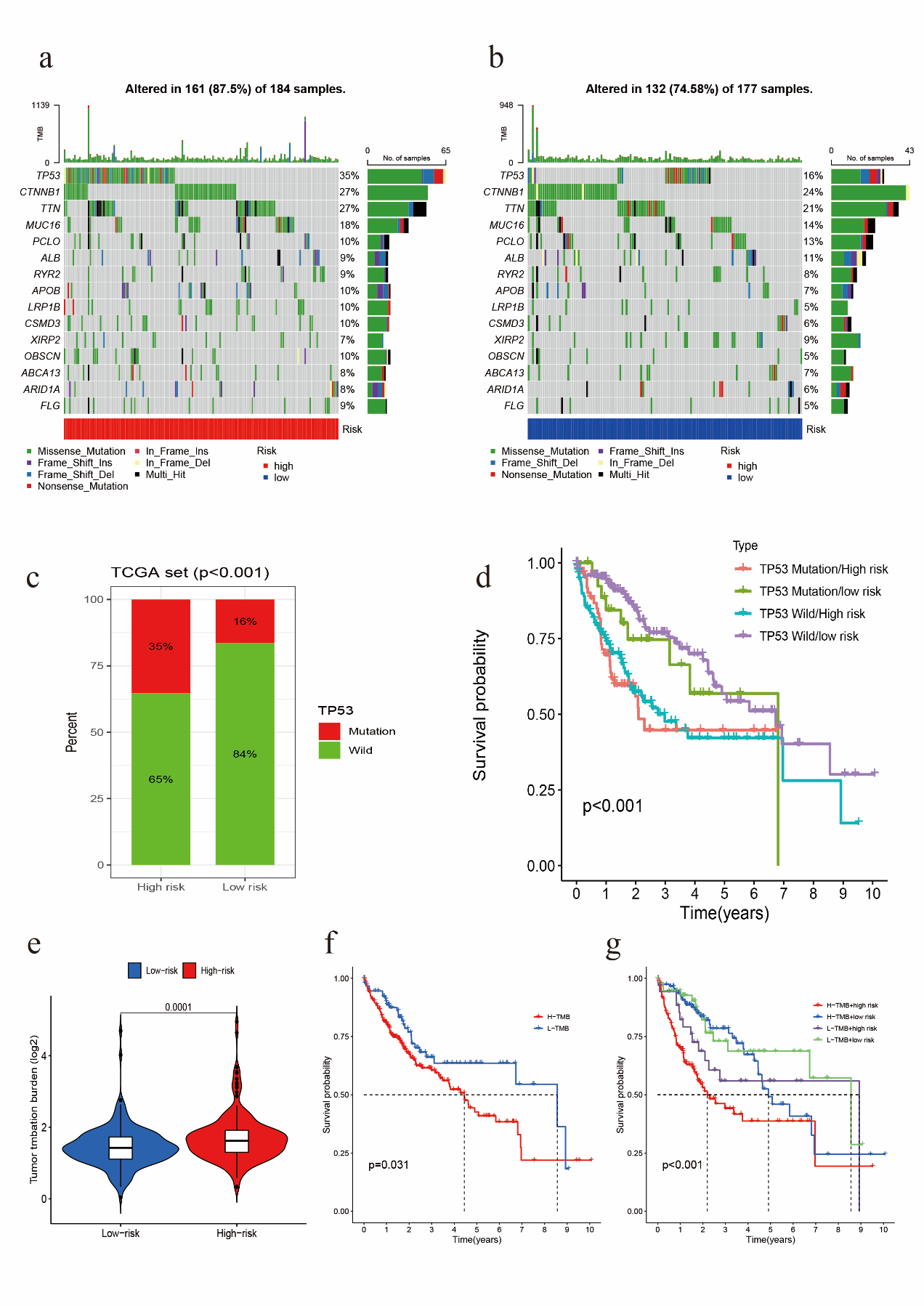

Supplement: Supplementary file 1 — Supplementary material 1 (DOCX 4000.5 kb) [file 10528_2024_10797_MOESM1_ESM.docx]
